# Powering the Environmental Internet of Things

**DOI:** 10.3390/s19081940

**Published:** 2019-04-25

**Authors:** Joshua Curry, Nick Harris

**Affiliations:** Department of Electronics and Computer Science, University of Southampton, Southampton SO17 1BJ, UK; jsc3g14@soton.ac.ukr

**Keywords:** Internet of Things (IoT), energy harvesting, novel energy harvesting hardware

## Abstract

The Internet of Things (IoT) is a constantly-evolving area of research and touches almost every aspect of life in the modern world. As technology moves forward, it is becoming increasingly important for these IoT devices for environmental sensing to become self-powered to enable long-term operation. This paper provides an outlook on the current state-of-the-art in terms of energy harvesting for these low-power devices. An analytical approach is taken, first defining types of environments in which energy-harvesters operate, before exploring both well-known and novel energy harvesting techniques and their uses in modern-day sensing.

## 1. Introduction

The Internet of Things is a constantly-evolving area of research, with many new applications and ideas being generated almost daily. From intelligent farming to smart devices in our homes, the IoT has affected almost every industry in our modern world [1].

One of the things that holds back this technology from achieving its full potential is power. Generally, IoT devices have reasonably low power consumption and are increasingly battery powered to enable operation in mobile or distributed applications.

Whilst providing a solid uninterrupted source of power, battery technology suffers from a range of drawbacks when used as part of IoT devices. These drawbacks can include the requirement of frequently recharging devices, potential pollution of the environment from damaged cells and degradation of the battery technology itself over a large number of charge cycles.

Energy harvesting is an attractive alternative to battery technology as it often mitigates most of these drawbacks, but often cannot be used as a swap-in solution to replace conventional battery-based systems. This is because energy harvesting supplies are very dependent on the environment in which they are situated and need to be managed intelligently alongside the load they are powering to allow for reliable operation [2].

For small, resource-constrained IoT devices, this creates an emphasis on power usage. Efficiency becomes vital, and energy scavenging becomes important to ensure long-term operation. There is no point harvesting energy and then wasting it on an inefficient system design. In an IoT setting, the efficiency of embedded systems has greatly improved in recent years, with advances in microcontroller [3] and low-power radio [4,5] technologies paving the way toward achieving deployable, fully-autonomous sensors [6].

Some systems have a necessary requirement for this full autonomy, especially in environments where changing batteries is not an option, for instance inside pipelines or nuclear installations. This leads to the idea of embedded sensing, or the installation of sensors in machinery or plants for the duration of its lifetime. This technique has been shown to be successful in infrastructure such as bridges [7].

Consideration needs to be given to the volatility of harvested energy and whether storage is actually required for the vast majority of environmental monitoring applications. Transient computing offers an attractive alternative to the long-term storage of harvested energy [8] and could negate the need to store harvested energy in large quantities locally in a sensor node. This technique utilises only instantaneous energy available from an energy-harvesting system, performing processing tasks when energy is sufficient and turning off when it can no longer sustain operation. A number of studies have investigated the use of this methodology in practice with promising results [9,10]. The largest advantage of this kind of technology is the removal of the need for significant local energy storage onboard a sensor node, paving the way for devices to be smaller and to offer less of a risk of contamination to the environment [11].

Although many comprehensive reviews are available in the field of environmental sensing as part of the Internet of Things [2,12,13], this review widens the scope to include less common ideas that may not be production ready, but look to offer great potential in the coming years. This review also proposes a comparison of such technologies with conventional solutions in terms of both energy output and environmental compatibility. These factors, combined with an indication of technology readiness, are intended to aid the readers in their choice of energy harvesting system and highlight up-and-coming technologies that may offer significant advantages in the near future.

## 2. Defining the Environment

The term “environmental monitoring” is very much dependent on one’s definition of the term “environment” and the individual constraints that each bring such as available forms of energy, containment issues and accessibility.

A very obvious split between environments is how definable the constraints of the environment are. In an outdoor setting, for instance a sensor node in the middle of a field, many constraints on the types of energy available are set by nature. Incident light, fluid movement and temperature are all dictated by the environment and are not available for the user to control [14]. Thus, any device harvesting energy from this environment must be able to cope with it’s unreliable nature. This is defined as an “uncontrolled” environment.

Uncontrolled environments surround us every day and can be broadly defined as outdoor areas where there is much variation on how much energy is present over time. As an example, uncontrolled environments could include regular farming/agricultural environments such as fields and rivers or harsher environments such as the sea or in space. It is worth noting that uncontrolled environments are not solely restricted to outdoor areas, as some other environments also experience the same large variations in net energy present over time, such as inside industrial greenhouses [15] or within large bodies of water [16].

In an indoor setting, the outlook is very different. Incident light is often defined by the amount of artificial lighting installed; temperature is often carefully controlled by temperature or HVAC systems; and fluid movement is often dependent on what processes and plant equipment are operating in the area. Systems operating in these kinds of environments often dissipate much energy, and this makes harvesting significantly easier for embedded devices as there is much more net energy available. This type of environment is defined as a “controlled” environment.

Controlled environments are broadly defined as indoor environments such as within buildings or monitored systems. Net available energy is very well defined, and often a reasonable estimate as to its magnitude can be obtained through mathematical means, using known data about objects such as machinery within that environment. However, controlled environments are not solely constrained to areas under shelter; the human body, for instance, could be considered a controlled environment. Temperature and pressure fall within very fine margins, and any system introduced into such an environment would be easily retrievable after use. This better defines a controlled environment, an environment where one or more sources of energy are well defined and any device could be easily categorised and retrieved after use.

In each different environment, different forms of energy harvesting can be used to process the available ambient energy into a usable source of power for sensors. Each different implementation is very application-specific, and thus, the whole system often needs to be designed around the source of energy for optimum operation. The use of energy already in an environment is termed “energy scavenging” [17].

Sometimes, in controlled environments, not enough energy is available for scavenging. Envisage a sensor node on the floor in the middle of a temperature-controlled warehouse. It is likely dark; there are no particular gradients of temperature or any large movement of fluid or air around from which to scavenge. In these circumstances, it may be required to inject energy from which the device can scavenge. This could be as simple as the installation of artificial lighting, which could be harvested using photovoltaic technology or could be as advanced as injecting an RF field across the room. This system of putting energy into the environment is defined here as “energy injection”.

These three discrete circumstances indicate that any energy-harvesting solution needs to be precisely tailored to its intended environment, together with a matched energy budget to ensure successful operation.

## 3. Sources of Energy

**Figure 1:**
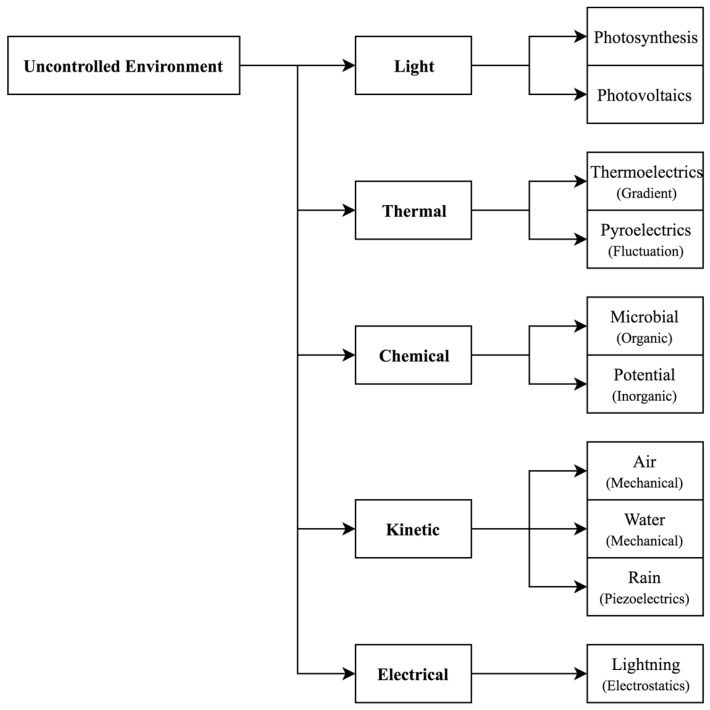
Examples of energy sources available in uncontrolled environments, which can be harvested using a variety of different techniques.

**Figure 2:**
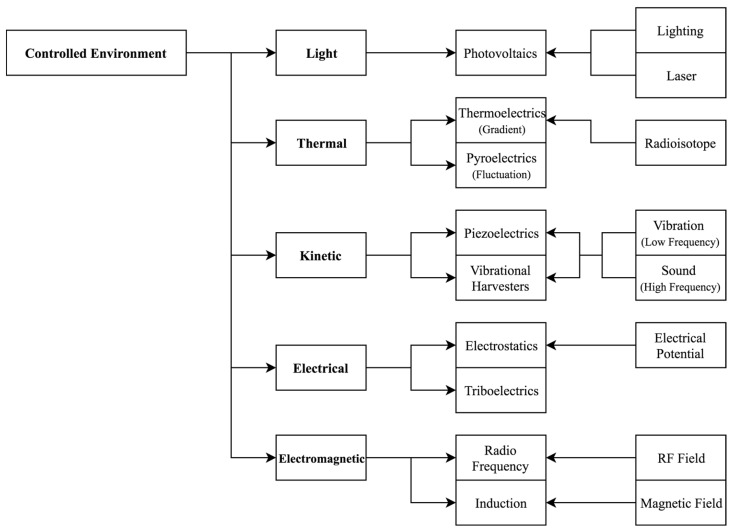
Examples of energy sources available in controlled environments. Although many energy sources are common to both types of environment defined, there are some important differences.

Figure 1 and Figure 2 present an overview of the common types of energy sources available in the environments defined above. This section explores these types of energy and indicates a range of technologies that are able to harvest from them. An outlook is provided for technology readiness in both controlled and uncontrolled environments, and sample figures for demonstrated outputs are given.

### 3.1. Light

Ambient light is a very predictable source of energy and is almost always present in both controlled and uncontrolled environments. Despite this, it is not a universal solution for the extraction of energy, as quantities of energy available vary vastly depending on the source of incident light.

Possibly the only source of readily-available natural light available is sunlight, and the harvesting of such energy and transforming it into electricity encompasses an entire field of science [2]. For uncontrolled environments, which are typically outdoor, this allows for the relatively easy collection of vast amounts of energy, and recent studies have implemented photovoltaic technology for the monitoring of many environmental parameters.

In an uncontrolled environment, available light is very predictable due to the known variations in sunlight intensity over both a typical day and over entire seasons. This repeatability can be exploited to improve the efficiency and thus reduce the overall amount of energy needing to be sourced. This has been demonstrated by recent studies that have shown the effective use of machine learning techniques to predict available solar energy [18]. Although these kinds of scheduling techniques have been available for a number of years and consist of hard-coded [19] or statistical techniques [20], recent developments in microprocessor technology have enabled the development of much more complex algorithms for energy prediction that incorporate machine learning techniques [21].

#### 3.1.1. Photovoltaics

Photovoltaic systems are perhaps the most prevalent energy-harvesting source used in the current age. From calculators to road signs, this staple form of energy makes use of the predictable nature of light sources, both natural and artificial.

There are many different types of photovoltaic technologies, but those utilised the most extensively in IoT applications are monocrystalline and polycrystalline solar cells [22]. Monocrystalline solar cells are more efficient at between 17% and 18% under outdoor conditions, however are typically more expensive due to smaller tolerances in the manufacturing process. Polycrystalline cells are slightly less expensive to fabricate, and this is reflected in their wholesale price, but offer a much lower efficiency at 12–14% [22]. Although many other fabrication methods have become available in recent years such as thin film and third-generation [23] solar cell technologies, which can reach efficiencies of over 40% [24], none have yet to match the price vs. efficiency balance offered by these two technologies. A particularly exciting area of photovoltaic research is that of organic solar cells. These types of cells utilise organic material in combination with a polymer base [23], paving the way towards more sustainable photovoltaic technology, which could one day be built on biodegradable substrates [25]. This kind of technology shows great promise for truly environmentally-compatible sensing systems with a minimal pollution footprint, albeit that it is currently a few years from matching the performance of conventional solar cells, with state-of-the-art efficiencies of around 10% [26].

All of these technologies require impedance matching to ensure that devices are always operating at their maximum power point. This is most commonly achieved using maximum power point tracking [27]. This technique can be implemented in many different ways, most commonly as part of a system-on-chip, for example a BQ25504 energy-harvesting IC [28]. Some studies have shown that modelling algorithms can be refined to optimise this technique in practice. This has allowed for the creation of MPPT systems with very low energy consumption, which maximise the active time of photovoltaic cells [29], and also systems that are heavily optimised for IoT applications [30].

In uncontrolled environments, the widest use-case for photovoltaic technology is as part of monitoring systems for precision agriculture. Due to the outdoor nature of these implementations, a stable, predictable power source is available at any time during the day. Many studies have shown the effective use of photovoltaic technology for these kinds of applications, with wireless sensor systems being developed that can effectively manage farmland irrigation [31], monitor glaciers [32] and measure water quality at sea [33].

These use-cases provide insight into the use of photovoltaic technology practically as part of a functioning network. However, they often utilise large photovoltaic cells in combination with bulky energy-storage mechanisms such as lead-acid batteries for reliability in an uncontrolled environment. This raises a question of energy budget; how much energy a sensor needs to operate and how this affects the design of such systems.

**Figure 3:**
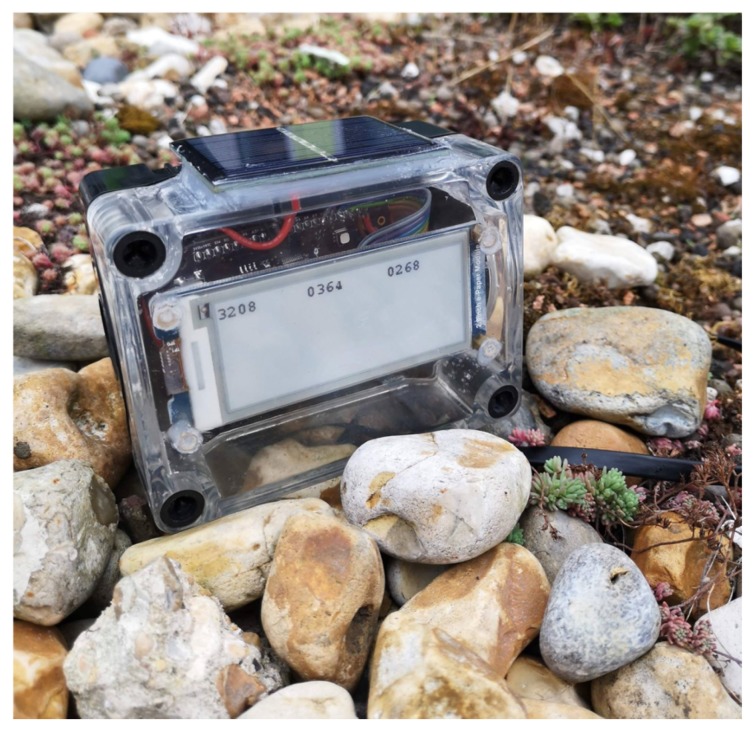
Photovoltaic technology provides a staple source of energy for a variety of energy-harvesting systems and is easy to implement as an addition to existing projects. This photo shows polycrystalline photovoltaic technology in use on an embedded sensing platform [36].

Some studies have shown that this technology can be miniaturised and still operate reliably, enabling battery-less operation [34,35]. This brings with it numerous advantages, increasing environmental compatibility by reducing the risk of contamination, increasing longevity by removing the limitation of charge-discharge cycles and reducing the overall cost of the node. Figure 3 shows an example photovoltaic system designed to experiment with this concept as part of a sensor network [36].

In controlled environments, sensors often co-inhabit industrial spaces with existing technology, making the need for miniaturisation much higher. In addition to this, the amount of available power under artificial lighting is typically 10-times less than under direct sunlight [37], making the design of such systems much more challenging, as more energy needs to be harvested in a smaller surface area.

Under indoor conditions, the efficiency of monocrystalline and polycrystalline photovoltaic cells drops drastically, with monocrystalline cells achieving just 1–3% under artificial lighting [38]. This makes the use of alternative photovoltaic technology more attractive under these conditions, with newer photovoltaic technologies able to achieve up to 10% efficiency under similar conditions, giving a demonstrated power output of up to 100 μW cm^−2^ [39]. However, these technologies are significantly more expensive, making them difficult to utilise at scale in a sensor network.

In direct sunlight, approximate outputs from monocrystalline and polycrystalline photovoltaic technology broadly vary around 50 mW cm^−2^ [37] This makes these technologies ideal candidates for many sensing applications in both controlled and uncontrolled applications, either directly or through a super-capacitor charging system.

A downside of the use of photovoltaic technology is the accumulation of dust and other opaque material on the surface of the cell over time. Mainly an issue in uncontrolled environments, this material can lead to a large degradation in the energy output of a photovoltaic cell and hence can reduce the lifetime of any sensing system dependent on it. Whilst other more unavoidable factors such as plants growing over the cell pose similar issues, the removal of dust and cleaning of photovoltaic systems has been the subject of various research as part of solar farms for power generation. Whilst simple mechanical solutions such as a mechanical wiper can be utilised in these larger power systems [40], on the scale of a small sensor, there simply would not be enough energy available to operate such a mechanism. A solution to this could be the use of electrodynamic screens to clean photovoltaic systems [41]. These solutions utilise a high electrical potential to electrostatically remove dust from photovoltaic devices and show great potential to be miniaturised for sensing devices due to their low power consumption and flexibility compared to other devices [41].

#### 3.1.2. Photosynthesis

Photosynthesis offers a viable alternative to solar cells for harvesting power from incident light. Typically utilising a biological or electrochemical reaction in combination with incident light to liberate electrons, [42], as a technology it is still very much in its early stages, but shows great promise for use as part of sensor systems in the coming years.

**Figure 4:**
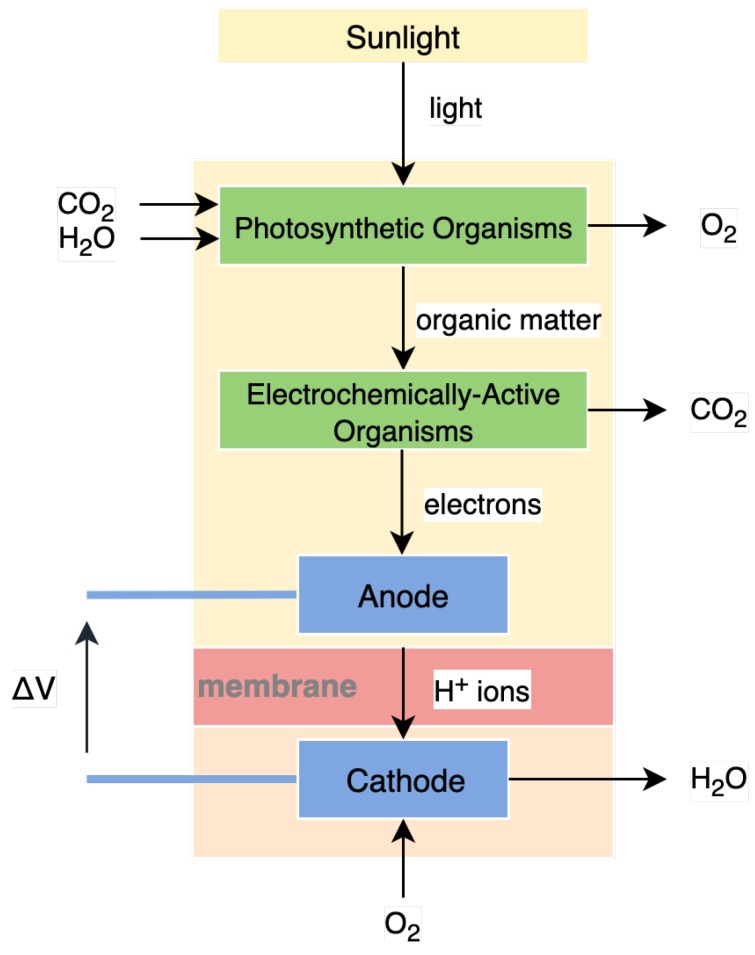
A model of a microbial fuel cell showing the process of the liberation of electrons; adapted from [42].

Figure 4 shows a block diagram of a typical photosynthetic microbial fuel cell, proposed by Strik et al. in 2011 [42]. In this example model, solar energy from the Sun in combination with carbon dioxide from the atmosphere and water allow photosynthetic organisms to produce organic material and oxygen. Electrochemically-active bacteria oxidise this material at the anode of the fuel cell, leading to the release of positive ions and electrons. These ions traverse a thin membrane between the anode and the cathode, reducing oxygen in the substrate back into water at the cathode, a process that utilises electrons. This biological process releases electrons at the anode, and utilises electrons at the cathode, producing a potential between them. This process can be utilised for energy harvesting [42].

Many different types of photosynthetic microbial and electrochemical fuel cells are the subject of current research [43], utilising different bacteria and algae to implement electron transfer [42]. In uncontrolled environments, an almost ubiquitous presence of light grants this technology much potential, possibly as an alternative for conventional photovoltaic systems.

In recent years, many studies have investigated the miniaturisation of microbial and electrochemical fuel cell technology [44,45,46] to smaller volumes than ever before, increasing the viability of the technology for use in small-scale systems. However, as a technology, it remains confined to use within the laboratory with only one or two studies characterising its use in real-world conditions [47] in combination with energy-harvesting circuitry.

Power output for this type of energy-harvesting technology varies from 10–40 μW cm^−2^ [47,48], making it a viable alternative to photovoltaic cells under some circumstances. With many benefits and better environmental compatibility compared to photovoltaic solutions, this could be a viable alternative for sensing in uncontrolled environments [42]. Although not widely utilised as part of sensing systems at present, in future years, this technology could find great use implemented in tandem with embedded devices.

A 2018 study showed that in an environment where energy injection is a viable solution, a control loop could be developed between sensing devices and a controllable energy source, injecting more energy into the environment when required by the end device [49]. This provides an optimal solution where energy input is managed intelligently, reducing waste energy dissipated into the environment, whilst ensuring that adequate energy is always available for end devices.

#### 3.1.3. Energy Injection

Where sufficient light energy is not present to utilise these methods of energy harvesting, there is potential to inject it. Only easily possible in controlled environments, light can be injected into the environment either by use of conventional sources, for instance artificial light, or by laser. Termed “optical energy transfer” [50], this technology utilises a source of high-intensity light and typically a transfer medium such as fibre optics to maximise power transfer. This technology is not typically utilised for embedded sensors, but could provide a solution for sensing at a distance by remote power utilising light energy harvesting methods in combination with a high intensity light source.

### 3.2. Thermal

Thermal energy harvesting has been available for embedded systems for a almost 70 years and has found home in a wide variety of technologies from large-scale satellites to small sensors.

#### 3.2.1. Thermoelectrics

**Figure 5:**
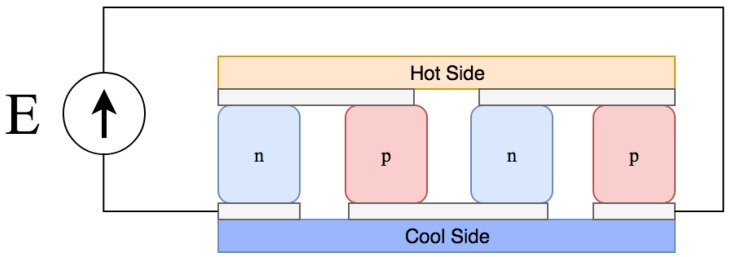
Thermoelectric generator system diagram.

Thermoelectric devices generate energy when a thermal gradient is placed across them. Typically consisting of semiconductor material with a high electrical conductivity and low thermal conductivity [51], this technology utilises the Seebeck effect [52] to generate a potential. A typical schematic of a thermoelectric device can be found in Figure 5.

Due to the necessity for the existence of a temperature gradient, typically, thermoelectric devices have not seen much use in uncontrolled environments. Typically, the only readily-available natural source of temperature gradient is incident solar energy, and that can be more efficiently harvested utilising photovoltaic technology. Some studies have investigated the use of thermoelectric technology as an alternative to photovoltaics for rooftop energy generation [53]; however, energy outputs were found to be much lower than equivalent solar technology of a similar area.

On the smaller scale of sensor devices, however, studies have shown that gradients between surfaces heated by the sun and the ground can be effectively utilised to provide enough energy to power sensors [54]. A recent study utilising the temperature differential between a light-absorbent panel and buried heat sink [55] also showed that this implementation can generate a reasonable energy output at night. This was due to a reversed thermal gradient when the air temperature became less than the temperature of the ground. Through harvesting both halves of this diurnal cycle, substantially more energy can be harvested from the system, with the study demonstrating that the energy generated from this reverse gradient at night can be as high as that generated during the day.

In controlled environments, waste energy is typically very common, and this provides an easy environment for thermoelectric harvesting. Many studies have shown the use of thermoelectric technology for telemetry in industrial environments, through the use of energy dissipated by machinery [56].

One potential disadvantage of this technology is the need to bring the maximum thermal gradient across the junction in order to make use of all energy present. This requires very heat-conductive materials to conduct energy quickly across the thermoelectric device and also adequate heat sinking on the opposite side to create the gradient. Insufficient optimisation of these parameters can lead to a lower efficiency.

This need for thermal efficiency can result in thermoelectric harvesters being quite large compared to alternatives. PCB fabrication techniques could provide a solution to this, with studies showing that aluminium-core [57] techniques can maximise power transfer while miniaturising the total device footprint.

In recent years, significant research has been conducted into wearable thermoelectric technology, powering sensors for the monitoring of bodily metrics. These types of energy harvesters could be considered to be operating in a controlled environment, as the temperature gradient between human skin and the air is relatively constant. Studies have shown that these types of thermoelectric generators can be miniaturised for use in this context [58], allowing devices to be smaller and less intrusive to the user.

In these types of wearable applications, thermoelectrics have been shown to provide up to 20 μW cm^−2^ [59] of usable energy during operation. In comparison with solar applications, a temperature gradient of 10 °C can produce up to 1.6 mW cm^−2^ under optimal conditions. This makes them a viable alternative to photovoltaic systems for systems that draw very small amounts of power.

Research has also been conducted into the use of thermoelectrics in harsh environments such as part of a car exhaust system, recovering energy from waste heat energy [60]. This system has been shown to be plausible and is the subject of current research by many major car manufacturers [61], but is still very much in the concept stages. In current research, it is subject to many drawbacks such as a low efficiency, high cost and lower weight-to-power ratio compared to other harvesting solutions [62], and hence, alternatives such as low pressure turbines are being considered as an alternative [63].

#### 3.2.2. Pyroelectrics

Pyroelectrics utilise the fluctuation of temperature gradients to generate energy [64]. This is useful in a variety of environments where temperature and pressures fluctuate rapidly, such as outdoors in uncontrolled environments and nearby machinery in controlled environments [65].

A high thermodynamic efficiency [65] and reduced heat sinking requirements make for a much smaller footprint than thermoelectric alternatives, and current studies have demonstrated thermodynamic cycle frequencies of the order of 1 Hz and material-level power densities of the order of 100 mW cm^−3^ [66].

However, as with thermoelectric technologies, the output power is dependent on the amount of thermal gradient brought across the device and also in this case with the frequency of temperature variation. Temperature variations in the order of Hertz are difficult to generate, especially in industrial environments where heat generation is typically constant. However, where such variations do not exist, studies have shown that they can be generated by connecting and disconnecting a pyroelectric material from an energy source and sink. This has been shown to be possible utilising a micro heat-engine, where the expansion and contraction of a working fluid mechanically introduces this temperature variation [67], with a practical device producing a 3-μW power output. It is predicted that optimisation of this type of device could result in a predicted power output of up to 9 mW [67].

Other methods such as switchable liquid-based thermal interfaces also allow these temperature variations to be induced [66]. These methods utilise liquid-crystal technology to switch heat conduction paths between energy sources and sinks, through a pyroelectric material. Although still very much in the concept stages, these devices offer predicted energy outputs of up to 113 mW cm^−3^, which is many orders of magnitude greater than the energy generated by pyroelectric material exposed to a single heat-cool condition [68].

In uncontrolled environments, typical temperature fluctuations are only exhibited over long periods and hence cannot on their own provide usable power output from pyroelectric technology. Similar oscillatory technology must be used in order to induce a fluctuating temperature gradient across the pyroelectric material. Studies have shown the successful combination of pyroelectric technology as a supplement to an existing piezoelectric solution [69], enabling the harvesting of energy from multiple sources to increase reliability.

Success has also been had in making pyroelectric technology CMOS-compatible, so that it may be utilised onboard existing silicon solutions. A 2018 publication [70] found that functional pyroelectric films could be deposited in trench structures on-chip and provide a very large harvestable energy density of 542 J m^−3^ K^2^. Although in its early stages, this technology shows great potential as it can be easily integrated with existing silicon designs, enabling on-chip energy harvesting and sensing.

Pyroelectric harvesting methods have a much higher efficiency than their thermoelectric counterparts, however requiring some intelligent management in order to generate a usable potential. In existing implementations, pyroelectric harvesters have been shown to have a power density of 0.034 μW cm^−2^ [68] as part of wearable technology with low temperature variation frequency and up to 3 μW from a heat-engine-based thermal harvester.

#### 3.2.3. Energy Injection: Radioisotopes

Thermoelectric devices have long been in use in combination with energy injection from radioisotope sources as part of space vehicles. Undergoing initial development in the 1960s, radioisotope power sources still power many vehicles that we send to space, with varying levels of power output from large generators producing hundreds of watts [71] to miniaturised systems producing just a few milliwatts [72].

In controlled environments, such as within spacecraft, radioisotope thermoelectric generators have proven to be invaluable for long-term space missions, and this is partly due to their high reliability and stability. However, over time, their available power output does reduce due to degradation of thermoelectric devices under harsh radioactive conditions [73,74]. Methods such as using an inert cover gas [73] can be employed to reduce this impact on the thermoelectric material, increasing the longevity of these systems.

In uncontrolled environments on Earth, it is difficult to utilise this technology due to the obvious radiological safety issues. Using our previous definition of an uncontrolled environment, recovery of the end device may not be possible in all cases, and this could potentially lead to the release of radioactive material into the environment. Hence, radioactive thermoelectric generators are only typically present where their use can be tightly controlled.

Cardiac pacemakers used to utilise this technology up to the late 20th Century. Providing a long-term uninterrupted power source for a number of years, their use was widespread [75], but has more recently been discontinued in favour of new battery technology [76].

Currently, research is being conducted into powering pacemakers from temperature differences within the human body itself, offering a much safer solution in comparison to the radioisotope thermoelectric generators of the past. A study of 2016 designed a thermal energy harvesting power supply for these types of systems, allowing a pacemaker system to make use of voltages as low as 40 mV outputted by a thermoelectric generator under small temperature gradients [77].

Radioisotope as an energy injection source can also be used to power other non-thermoelectric harvesting methods. A study of 2002 suggested the design of a Stirling-engine-based system for thermal energy harvesting from a radioisotope source [78]. This was made possible because of the large amount of thermal energy available from such a source, making this concept harder to replicate with smaller sources of thermal energy.

### 3.3. Chemical

#### 3.3.1. Microbial

Microbial power sources for embedded sensors utilise biological reactions to “digest” reactants, producing an electrical potential in the process [43]. Unlike photosynthetic cells, covered in Section 3.1.2, these types of fuel cells can operate in environments where there is no light present, making them useful for extended, bio-compatible deployments.

In controlled environments, microbial fuel cells can provide a stable source of energy when utilised as part of wastewater treatment. A 2011 study [79] developed a sensor network for wastewater monitoring, showing the potential of utilising Granular Activated Carbon Single-Chamber Microbial Fuel Cells (GAC-SCMFCs) to power the network. The use of this technology as part of controlled environments has many advantages such as a high power density of up to 7 W m^−2^, and relative self-sufficiency, enabling systems to be embedded in part of a process system for the duration of its lifetime.

In uncontrolled environments, microbial fuel cells have proven to be a viable source of energy for deep-sea sensing. A 2015 study found that microbial fuel cells could be used to power a deep-sea sensing device [80]. With power outputs of up to 10 mW cm^−2^ at small depths of 5 m and up to 0.5 μW cm^−2^ of anode area at 1000 m, this technology shows great potential for use as part of deep-sea sensing exhibitions, as it makes use of a unique source of energy, one of very few available at such a depth below the sea surface.

Research has also been conducted into the use of microbial fuel cells on land. A 2013 study [81] showed a 3-mW cm^−2^ power output from a terrestrial microbial fuel cell, using soil as a substrate. Other studies of terrestrial microbial fuel cells have found power outputs of 0.31 mW for a small reactor fitting within a 10 × 10 × 10-cm^3^ cube [81].

A 2019 study showed the ability to combine one of these land-based MFCs, specifically a plant-based variant, with a microcontroller system to transmit temperature data. It was found that a power density of up to 3.5 mW cm^−2^ was able to be generated, more than satisfying the current demand of 0.35 mA required by the sensing system.

The use of this technology on land could enable many new sensing missions able to operate in new uncontrolled environments, specifically where solar radiation is not available. However, more study needs to be conducted into their longevity for powering sensors, as land-based MFCs do not experience the same replenishment of reactants as water-based systems.

#### 3.3.2. Chemical Potential

An alternative chemical method of energy harvesting to microbial fuel cells is electrochemical harvesting. This technology utilises the transfer of electrons by electrochemical means to effect a usable potential. Available for years, this technology essentially works in a similar fashion to battery technology, where chemical energy is transformed into electrical potential.

In recent years, much research has been conducted into the use of the natural corrosion of steel structures to produce energy. This would yield an almost limitless source of power for embedded sensors, which could be embedded within infrastructure for the duration of its lifetime. The corrosion of steel is at its root an electrochemical process, which is capable of releasing micro-energy, which can further be harvested [82].

Finding practical implementation as part of a number of sensing projects, the use of steel corrosion as a means of powering sensors has been present in modern-day research for a number of years [82,83]. Such systems show great potential for long-term monitoring of steel structures and could pave the way towards truly embedded sensing.

The natural corrosion of other metallic compounds can also be used for powering sensors. A 2012 study [84] constructed a “concrete battery” utilising magnesium and zinc electrodes embedded within a conductive cement composite. The study found that such a technique was capable of powering embedded devices from a generated electrochemical potential and also could be combined with a further graphite electrode to determine the proportions of aggressive agents in the concrete factor in its longevity.

A 2014 study [85] categorised a number of samples of “cement battery”, which had been soaked in seawater for two weeks to simulate operation in an uncontrolled marine environment. It was found that the power output from such a concrete battery was in the region of 200–500 μW dependent on the load resistance connected. This was further abstracted into a calculation of approximate energy storage capacity required to power a sensing system off such an energy-harvesting source.

The use of chemical potential in a number of practical sensing projects demonstrates its reliability as a source of power for sensing devices. This technology could be useful in many environments, both controlled and uncontrolled, where corrosion is present, including as part of infrastructure and marine applications.

### 3.4. Kinetics

#### 3.4.1. Fluid Movement

Many conventional mechanisms exist for air and water movement, the widest used of which is the conventional turbine. As with photovoltaic technology, these can be manufactured to take up a large area, hence producing a significant amount of energy [2], but on the small scale of environmental sensors, different approaches need to be taken.

Although microturbines have been proposed that could harvest this type of energy on the micro-scale [86], they have proven difficult to implement in uncontrolled environments. This is because typically with airflow systems, power density scales per unit area, whereas frictional effects on the device do not scale at the same rate. This means that such devices cannot operate at the lower airflow speeds typically found in environments. They also have a high degree of moving parts, which can easily wear out over time and require replacement. This restricts their size to larger volumes. Microturbine solutions in the order of 300 cm^3^ volume have been shown to produce up to 10 mW at a wind speed of 4.5 m s^−1^ [87].

In controlled environments, sources of fluid movement include the movement of air in ventilation ducts and the movement of liquids within piping. This provides a wide spectrum of sources of energy to harvest. In uncontrolled environments, air movement occurs naturally with the changing of weather, and hence, applications here can benefit from this type of harvesting technology also.

In this type of environment, microturbines do propose a viable solution, as there is significantly more energy available to harvest per unit area. A recent study [88] showed that soil moisture sensors can be self-powered by a microturbine device integrated into micro-sprinklers utilised as part of an irrigation system. This system makes use of the high water flow during irrigation periods to generate energy and shows an innovative method for integrating smart devices into irrigation systems.

**Figure 6:**
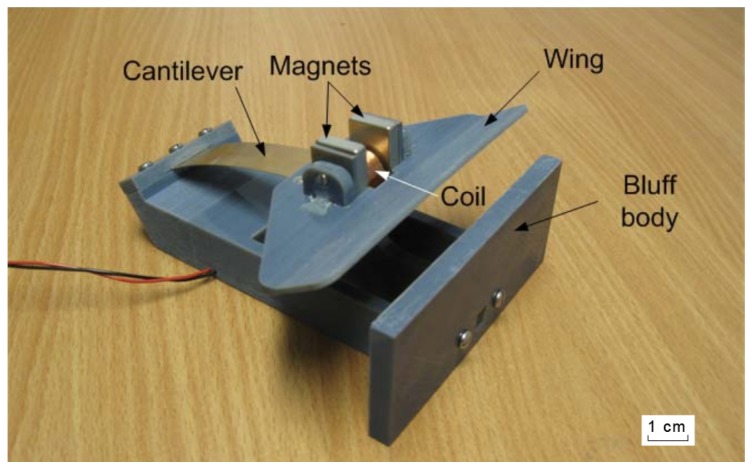
Horizontal airflow energy harvester [89].

Many small-scale fluid movement harvesters achieve energy harvesting through the use of an oscillating beam. This makes use of the vortex effect [89] when the movement of air passes over an object that resists airflow. The movement of this air causes this object, combined with an elastic mount, to begin oscillating, and these oscillations can be turned into electrical energy. In the device shown in Figure 6, this utilised a small electromagnetic coil and permanent magnet, but other studies have suggested the potential to utilise piezoelectric material for this purpose [90,91].

This resonating harvester design can be miniaturised onto silicon [92] and is possible to stack as part of a multi-harvester system to generate larger amounts of energy in a smaller space. This study reported a maximum energy output of 42.2 μW at 20-m s^−1^ air velocity.

As a moderate-scale technology, fluid movement harvesting in air has been demonstrated to provide up to 90 μW at moderate velocities of 2 m s^−1^ [89]. Although able to harvest significantly more energy at higher velocities, this comes at the detriment to efficiency. Such oscillating beam harvesters may experience damage at extremely high velocities as larger oscillations may become damaging to the device, if they exceed the elastic limit of the harvester. If this limit is not reached, however, the lifetime has no limitations.

#### 3.4.2. Piezoelectrics

Piezoelectric harvesters are utilised as part of a large proportion of energy-harvesting solutions. Making use of doped Lead Zirconate Titanate (PZT), which is a piezoelectrically-active material, these types of harvesters are mostly used to collect mechanical energy and translate it directly into electrical potential. Impulsive transients are required in order to induce charge separation with piezoelectric systems, and as such, many harvester designs translate linear energy such as a constant airflow or force into vibration, which can then be harvested piezoelectrically [93].

A large area of piezoelectric-based harvesting is air movement, as discussed above. This application typically utilises a mechanical system to interact with a piezoelectric compound either through impact or bending, to produce charge separation and therefore energy [94]. Piezoelectric solutions have many advantages over electromagnetic harvesters for mechanical applications as they typically do not require as much lateral movement to harvest energy, allowing them to be significantly smaller. Piezoelectric devices can also be manufactured using thick-film technology [95], enabling their use in a variety of custom applications such as vibration harvesting for helicopter applications [96].

Success has also been found in utilising piezoelectric technology to harvest energy from repeated movement. Some studies have found that integrating such technology into clothing and footwear can lead to usable energy output [97], with some studies harvesting energy outputs of up to 8.8 mW from the human walking cycle [98]. This study utilised a harvester built into a shoe, but other studies have also investigated the potential of harvesting energy from the other side of the walking interface, utilising piezoelectric devices embedded in floors and walkways [99]. Although an attractive source of energy, walking is inherently a very efficient process, and the extraction of energy from such a process through interactive floors and materials can lead to user discomfort [100]. As such, it is important to regulate the extraction of energy from this process to ensure that it does not affect the generation mechanism, otherwise people could avoid walking on the harvester, negating its purpose.

In uncontrolled environments, piezoelectric harvesters have been demonstrated as part of rain-energy harvesting systems. Energy output from such systems is proportional to many factors, such as droplet diameter and velocity [101], but studies have found that under optimal conditions, up to 12.5 mW of power output is possible from such technology [102]. Many different designs of harvesters for this type of application are available [103], and studies suggest that adequate optimisation of existing solutions could lead to a great performance improvement in coming years [104].

#### 3.4.3. Atmospheric Variation

In many uncontrolled environments, variation occurs naturally with changes in weather from day to day. This diurnal variation in temperature and pressure can yield usable power with novel research into atmospheric variation harvesters.

Such harvesters typically consist of a phase-change fluid in a sealed medium, in combination with a set of bellows or equivalent mechanism for harvesting the mechanical energy produced by the expansion and contraction of the fluid due to temperature differences. Studies commonly utilise ethyl chloride for this purpose due to its low boiling point of 287 K [105].

Studies have utilised this technology to implement energy harvesting from temperature variations in the environment. Such harvesters show great potential for the harvesting of energy in areas with a large diurnal temperature variation, with a 2015 publication showing up to 6 J of energy being achieved with a 23 K temperature change [105].

Atmospheric energy harvesting has been available for a number of years, first making public view as part of the “Atmos Clock” system. Another study has further refined this system by utilising bellows from the original clock design in conjunction with a brushless motor to generate energy for a wireless sensing system [106]. Although only yielding up to 21 mJ in a 24-h period, this technology certainly shows promise for long-term low-frequency solutions such as embedded sensors.

Across a 24-h period in uncontrolled environments, temperature variation is very slow, following a trend synchronised with the rise and fall of the Sun. In controlled environments, larger fluctuations can be seen, particularly as part of machinery and airflow systems. A 2017 study found that useful energy could be harvested from low-frequency temperature variations of 2 °C, producing 9.6 mW across a 24-h harvesting period [107].

### 3.5. Electrical

#### 3.5.1. Electrostatics

Electrostatic energy-harvesting systems, or “electret generators”, utilise a variable-capacitor-based structure in combination with mechanical energy, generating charge from motion between the two plates. Typically utilised for vibration energy harvesting, this solution can be fabricated on much smaller scales than conventional electromagnetic technology and operate at much higher frequencies.

This technology has been shown as part of a 1978 publication [108] to demonstrate energy outputs of up to 25 mW as part of a 6000-rpm rotating system, albeit with an active area of 730 cm^2^ [109]. Although impressive, this solution required an incredibly large active area, too large for any embedded sensor. As such, much modern-day research investigates the use of this technology as part of Micro Electro-Mechanical Systems (MEMS).

MEMS fabrication techniques have allowed such devices to be micro-machined, with a 2007 publication fabricating an MEMS electret generator capable of up to 5 μW of output [110]. The optimisation of these devices is very much a subject of current research, with some studies suggesting that a power output of up to 549 μW cm^−2^ could be harvested from rapid accelerations of 0.6 g from such devices [111], showing great potential for future implementable systems.

#### 3.5.2. Triboelectrics

Triboelectric harvesters utilise a very specific phenomenon called the triboelectric effect. This effect becomes apparent when certain materials are brought together and subsequently separated, leaving electric charge on the surfaces that were previously in contact [112]. This can be exploited to harvest energy from scenarios where mechanical movement is present, for instance as part of wearable technology.

**Figure 7:**
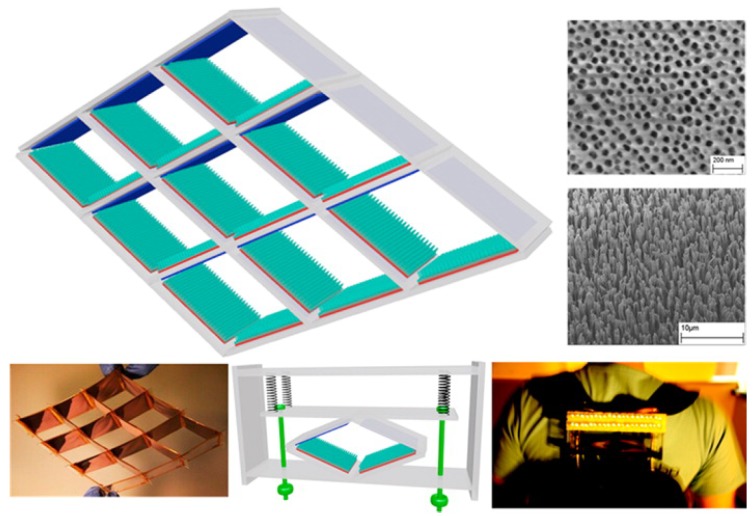
Triboelectric nanogenerator for harvesting ambient mechanical energy. Reprinted with permission from [113]. Copyright (2013) American Chemical Society.

This bringing together and separating of materials can be exploited in scenarios where linear reciprocal movement is present such as in the human walking cycle [113]. Utilising a precisely-designed mechanical harvester, depicted in Figure 7, a number of triboelectric regions can be brought together and separated by vibrations generated as part of walking. This system shows great potential for alternative mechanical energy harvesting, allowing small-scale generators with no need for bulky electromagnetic coils.

This technology could provide up to 3 mW cm^−2^ of electrical energy [113], which gives a larger energy output within a smaller footprint compared to other technologies.

### 3.6. Electromagnetic

#### 3.6.1. Radio Frequency

Radio frequency energy is widespread in both controlled and uncontrolled environments. Radio frequency energy harvesting is here differentiated from induction energy harvesting, as it does not rely on direct magnetic coupling between two coils and instead allows energy transfer over a longer distance [114].

In controlled environments, a much larger amount of radio frequency energy is available to devices than in uncontrolled environments. This is due to the presence of man-made wireless devices that emit such radiation, such as WiFi routers and sources of RF power injection. As such, many projects have attempted to exploit this to harvest energy, showing promising results. Publications have seen devices harvest WiFi energy in the order of gigahertz [115] and from cell-tower broadcasts in the order of hundreds of megahertz [116]. Networks have also been developed in modern research to utilise the power from other sensor nodes to harvest energy. Simultaneous Wireless Information and Power Transfer (SWIPT) networks can make use of RF energy-harvesting technology to create a single wireless system encapsulating both power and data [25]. This type of system shows great potential for use in both controlled and uncontrolled environments, however requiring intelligent resource management to ensure reliable operation [117].

A key defining factor of RF energy transfer technology is the design and implementation of antennas through which the energy is transmitted and received. These antennas can greatly vary the size of devices, as they need to be very well matched to the frequency of operation in order to enable high efficiency. Device placement and antenna directionality is also important to maximise the amount of transmitted energy coupled into the receiving antenna. Many historical studies have developed this area of RF technology for applications such as powering remote devices using microwave beams [118].

Over short distances, RF energy harvesting can provide significant levels of power transfer, in the order of tens of milliwatts within two metres of an RF transmission source [119]. This is useful for potential applications in controlled environments where power sources are nearby sensors, however making RF energy transfer difficult at longer distances such as those found in uncontrolled environments.

Many commercial ventures have attempted to capitalise on the wireless transfer of power utilising radio frequency energy transfer. An example of this is PowerCast, who offer the ability to charge modern-day devices wirelessly over reasonable distances from a set transmitter. Recently becoming available as embedded circuitry from component giant DigiKey [120], PowerCast utilises the 915-MHz ISM band to transfer energy over the air, with some embedded solutions providing up to 50 mA at 5 V to embedded devices [121]. This is, however, very dependent on the RF environment, and range can be somewhat limited by physical objects such as walls and furniture. Studies show that PowerCast technology can provide up to 16.115 mW at a 0.6-m distance with an appropriate antenna, but this drops off rapidly to 26 μW at a 4-m distance [122].

In uncontrolled environments, researchers have shown that low-frequency RF energy can be utilised over much larger distances compared to high-frequency counterparts such as GSM and WiFi [123]. A very exciting new development in wireless power transfer systems is the creation of a medium-frequency CMOS energy harvester [124]. This system offers the ability to embed a power source into any CMOS device, which can supply up to 10 μW and occupy just 0.54 mm^2^ in silicon. Although very small, this does not include the receiving coil, for which dimensions would need to be known to calculate power density. This solution offers an exciting application for the use of RF energy transfer in uncontrolled environments and could find application in many areas such as soil condition monitoring and embedded sensing.

#### 3.6.2. Induction

Electromagnetic induction has been available for years as a charging method for mobile devices, but it can also be utilised to power sensor devices. Utilising strongly-coupled magnetic devices, this method of power transfer can achieve significant magnitudes of power transferred, at reasonably acceptable losses. MIT researchers in 2007 developed a system that could transfer up to 60 W of RF energy at 40% efficiency across a 2-m air gap [125]. This research shows the potential of this technology for large-scale power transfer.

Induction energy transfer is not common in uncontrolled environments due to a lack of nearby power sources from which to transmit energy and is instead mostly bounded to controlled environments where electrical energy sources are widespread. However, it finds use in a number of more controlled environments where there is a larger energy transfer.

Another example of an enclosed system into which energy transfer is desirable is the human body. Multiple studies have shown that energy can be safely transmitted into embedded sensors in human tissue [126]. This offers a way forward to truly embedded sensors, rechargeable using a non-contact method for in vivo monitoring.

Companies such as Wi-Tricity [127] have utilised this magnetic resonance technology [128] for the large-scale charging of devices, from cell phones to electric cars. This method of energy transfer requires a much shorter range than the RF solutions above, but power transfer has been demonstrated in the order of tens of kilowatts [129].

A potential use case for induction energy is within enclosed systems such as nuclear reactors. In these types of controlled environments, systems must be environmentally isolated from each other to prevent the release of toxic material into the atmosphere. However, such systems often utilise metallic components that make induction difficult to achieve. Studies have shown that super-low-frequency induction can be utilised to reduce the dissipation effects of metal surfaces [130], increasing the use cases for this type of technology by expanding operating environments.

## 4. Discussion

Energy harvesters based on photovoltaics have been present in our modern world for years, finding their way into a variety of modern applications such as smart infrastructure [131], and continue to power remote systems reliably in combination with energy storage technology. Some novel methods in recent years have shown great increases in the available efficiency of such systems and highlight the potential that one day, such systems could be biologically compatible. Photosynthesis looks to be an exciting alternative to this technology, but at the time of publication remains unproven as a part of a real-world embedded energy-harvesting solution.

Thermal energy harvesters have seen large advancements in recent years, particularly in the region of pyroelectric systems. Conventional thermoelectric generators have been available since the mid-20th Century and provide consistent power to many projects both in industry and in space, in combination with radioisotope thermal injection. Although reliable, these devices require a significant heat sinking apparatus to be utilised with large temperature gradients and offer limited performance with smaller ones. Pyroelectric devices allow energy to be extracted far more efficiently from heat sources, with a much smaller footprint than conventional thermoelectric devices. Although requiring significantly more technical complexity to implement, these devices as CMOS-compatible systems could offer truly embedded energy harvesting in the near future.

Mechanical energy harvesters have seen a large shift towards the micro-scale in recent years. Whilst wind generation has been a stable source of energy for society for many decades, we are only just seeing the start of micro-scale energy harvesters for sensing applications in this field. Whilst very reliable and with large power outputs in large air velocities, these types of harvester can easily suffer from fatigue if the correct materials are not considered, but could provide a very usable source of energy for sensing technology in the near future.

Chemical means of energy harvesting are only just starting to be miniaturised for use on embedded sensors. Studies have seen them utilised in both industrial processes and deep at sea, both areas where local power is difficult to obtain. Particularly in the area of deep-sea exploration, microbial energy harvesting is one of the only technologies enabling autonomous operation for sensors in this type of environment, and hopefully, they will continue to be miniaturised and studied so that they can be utilised on long-term sensing missions. The use of corrosion chemistry to monitor concrete reinforced structures is particularly fascinating, and it is anticipated that these also will be deployed into commercial sensing use, enabling sensors to be truly embedded within infrastructure.

Novel mechanical harvesters, utilising piezoelectrics in combination with impulsive transients such as rain and vibration, offer a unique way to harvest otherwise waste energy. Whilst their high potential output and short pulse width make energy collection difficult, they provide efficient access to mechanical energy in a way no other harvesters can, and it is anticipated that their use will be explored as part of more linear energy-harvesting systems to maximise available energy outputs. Pressure change harvesting is one of the newest areas of research and has much potential if pressure variation can be maximised. In the future, this technology combined with extremely low-power devices could pave the way toward truly autonomous sensing in uncontrolled environments.

Radio frequency and induction technology offer both large-scale and large power transfer solutions, albeit at only small efficiencies. Although not finding their place in the energy-harvesting domain as part of environmental sensing at this point, it is expected that novel new technologies and miniaturisation will allow it to be utilised in more uncontrolled environments, enabling low-power sensing systems to be embedded below ground or within infrastructure.

**Figure 8:**
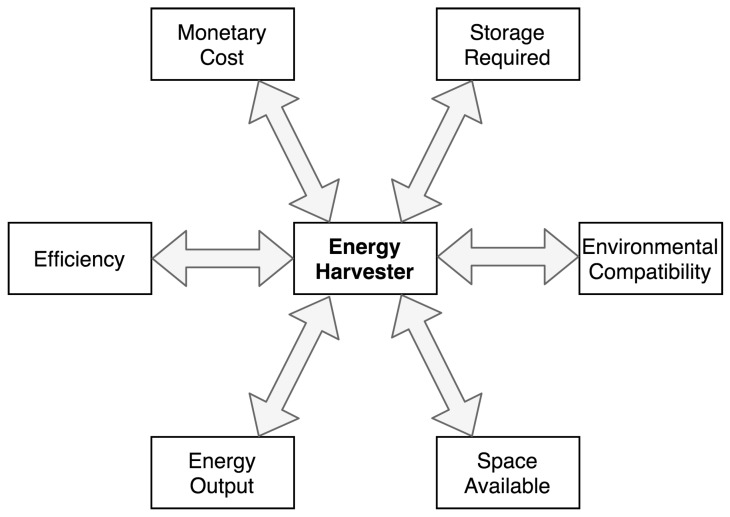
Examples of design considerations for energy-harvesting systems.

This study has categorised a variety of energy-harvesting technologies. Some devices output a net energy per unit area and some per unit volume. This is a vital consideration to make when designing a sensor device as some technologies, for example photovoltaics, have a negligible thickness, whereas some other devices such as fuel cells or fluid movement harvesters can occupy much more space volumetrically. Hence, it is important to compare the amounts of energy required and the volumetric space available and select an appropriate harvester accordingly. Energy harvester selection could be said to be a balance between many areas, and Figure 8 attempts to categorise these main design decisions into a single diagram.

The overriding requirement of any energy-harvesting system is the existence of energy in a form that is harvestable. This is very environmentally dependent, with the categorised environments discussed in Section 2 each providing different prevalent forms of energy that is able to be harvested. Although energy availability is important, the practical implementation of these types of devices is also subject to other important factors.

Monetary cost is a key consideration for any type of energy harvester. The best solution for systems with multiple end devices will not always be the least expensive, but a certain consideration should be made of the cost of deploying technology that is currently only confined to the laboratory into working environments. A key place where this takes effect is the preference of the use of photovoltaic technology over microbial technology at the current time. Photovoltaics are inexpensive and easy to replace, whereas photosynthetic fuel cells can be expensive and hard to implement without large cost, as this is still a laboratory-based technology.

Storage capacity required should be calculated and investigated as part of the sensor design process. Some energy-harvesting technologies such as piezoelectric and pressure change systems require large accumulations of energy in order to run embedded devices, whereas other solutions such as air movement harvesting require smaller accumulations as they typically provide more power.

Environmental compatibility is a key issue, especially in uncontrolled environments. New energy harvesting systems such as organic photovoltaic cells and microbial fuel cells could pave the way toward truly environmentally-compatible systems, but current-day sensor design processes should consider the effect of their components on the environment, during and after the projected mission.

Space available is a key parameter for any system. It would not make sense to implement a relatively large energy harvester if available space was limited, and micro-sized solutions should be considered instead.

Efficiency has not been covered in great depth by this study, but is a key design choice when implementing an energy-harvesting solution. If very small amounts of energy are available in an environment, as has been seen with small-scale systems such as pressure variation and fuel cell technology, the efficiency of the system should be optimised to ensure the smallest possible energy loss. With controlled environments and energy transfer technologies, where much power is available, efficiency is less of a factor, and that can be seen in many implemented RF power distribution systems.

Energy output is the most key component of the entire system. An implemented solution needs to provide enough energy to power devices connected to it, otherwise the mission would not be able to function. With advances in low-power microprocessor and sensing technology, power requirements for embedded systems are decreasing, but consideration should be made as to the system’s energy budget and how energy-harvesting systems contribute to it.

Table 1 shows a compilation of demonstrated energy sources described in Section 3. Whilst it attempts to show a detailed comparison of each energy source discussed against alternatives, it is worth noting that it is not a definitive list.

This paper has discussed a variety of available solutions for harvesting energy in both controlled and uncontrolled environments. The ability to be energy-neutral as a sensor enables a wide range of potential applications to be explored in the areas of communications and computing. A particularly exciting field that is enabled by integrating multiple energy-harvesting solutions discussed in this paper is multi-source energy harvesting [136,137]. This area of research integrates multiple instances of harvesting technology into an end device, allowing it to harvest significantly more energy than would be available utilising just one source. This can mitigate many of the issues that arise through a lack of energy in one area and greatly contribute to the longevity of end devices.

Another area that is enabled by the inclusion of these technologies is the development of energy-aware wireless protocols and systems. Multiple studies have shown that wireless protocols can be developed to both use less energy during transmission and reception [138] and include adaptive protocols that work alongside energy-harvesting technology to manage network connections to end devices [139,140].

All of these solutions show exciting potential for revolutionising the longevity and wireless communication characteristics of IoT devices, allowing an ecosystem to be created where end devices can be deployed for extended periods and intelligently managed for long-term data collection.

## 5. Conclusions

As the Internet of Things becomes more ubiquitous and the types of applications become more imaginative, it becomes necessary to be more imaginative in the powering of any autonomous devices. In this survey, we have briefly discussed traditional energy-harvesting solutions for remote devices in various environments, but we have also tried to extend the discussion to more unusual solutions that may be less developed, but may offer long-term potential. It has become apparent that the definition of environment becomes key to defining the correct solution for a particular application, but equally important are the definitions of the system power budget, as using energy efficiently is as important as providing it.

As an extension of these ideas, where an appropriate amount of energy is not available for extraction from a given environment, energy injection techniques can be employed to provide it, enabling sensing systems to operate in a variety of environments in which natural sources of energy are not present or are not significant enough. Currently as an example, it would appear that for traditional outside applications, the most common solution is to use a photovoltaic system, as this is effective and widely known, but has drawbacks that affect its efficiency in the longer term, such as vegetative cover or dust accumulation. Thus, alternative technologies such as pyroelectric and pressure (temperature) change systems may be more effective over the longer term as these deficiencies are more controllable. However, there is no one complete panacea, which brings us back to the importance of correctly assessing the environment for a given mission. However, there are many technologies being developed that offer promise in the next few years, and the choice for system designers will open up significantly. However, with the plethora of systems that are likely to be deployed, technologies that are in themselves non-polluting or environmentally friendly will become more significant.

## Figures and Tables

**Table 1:** A compilation of demonstrated energy outputs from sources discussed.

Harvester	Demonstrated Power Output	References
Photovoltaic (Outdoor)	50 mW cm^−2^	[37]
Photovoltaic (Indoor)	50 μW cm^−2^	[37]
Photosynthesis (Lab)	10–40 μW cm^−2^	[47]
Thermoelectrics	20 μW cm^−2^	[59]
Pyroelectrics (lab)	8.64 μW cm^−3^	[132]
Microbial	3–700 μW cm^−3^	[79,133]
Chemical Potential	3 mW	[82]
Air Movement	6 μW cm^−2^	[89,94]
Pressure Variation	15 μW cm^−2^	[105]
Piezoelectrics	12.5 mW	[102]
Triboelectrics	3 mW cm^−2^	[113]
Electrostatics	12 mW cm^−2^	[134]
Radio Frequency	10.3 μW system	[124]
Induction	70+ μW cm^−2^	[135]

## References

[1] Xu L.D., He W., Li S. (2014). Internet of Things in Industries: A Survey. IEEE Trans. Ind. Inf..

[2] Prauzek M., Konecny J., Borova M., Janosova K., Hlavica J., Musilek P. (2018). Energy Harvesting Sources, Storage Devices and System Topologies for Environmental Wireless Sensor Networks: A Review. Sensors.

[3] Botman F., de Vos J., Bernard S., Stas F., Legat J., Bol D. Bellevue: A 50 MHz variable-width SIMD 32bit microcontroller at 0.37 V for processing-intensive wireless sensor nodes.. Proceedings of the IEEE International Symposium on Circuits and Systems (ISCAS).

[4] Raza U., Kulkarni P., Sooriyabandara M. (2017). Low Power Wide Area Networks: An Overview. IEEE Commun. Surv. Tutorials.

[5] Harris N., Joshua S.C. (2018). Development and range testing of a LoRaWAN system in an urban environment. Int. J. Electron. Commun. Eng..

[6] Kroener M. Energy harvesting technologies: Energy sources, generators and management for wireless autonomous applications. Proceedings of the International Multi-Conference on Systems, Signals Devices.

[7] Ha D., Kim D., Choo J.F., Goo N.S. Energy harvesting and monitoring using bridge bearing with built-in piezoelectric material. Proceedings of the 7th International Conference on Networked Computing.

[8] Merrett G.V. Invited: Energy harvesting and transient computing: A paradigm shift for embedded systems?. Proceedings of the 2016 53rd ACM/EDAC/IEEE Design Automation Conference (DAC).

[9] Ahmed F., Ahmed T., Muhammad Y., Le Moullec Y., Annus P. (2016). Operating Wireless Sensor Nodes without Energy Storage: Experimental Results with Transient Computing. Electronics.

[10] Ahmed F., Kervadec C., Le Moullec Y., Tamberg G., Annus P. (2018). Autonomous Wireless Sensor Networks: Implementation of Transient Computing and Energy Prediction for Improved Node Performance and Link Quality. Comput. J..

[11] Wanger T. (2011). The Lithium future—Resources, recycling, and the environment. Conserv. Lett..

[12] Tang X., Wang X., Cattley R., Gu F., Ball A.D. (2018). Energy Harvesting Technologies for Achieving Self-Powered Wireless Sensor Networks in Machine Condition Monitoring: A Review. Sensors.

[13] Akan O.B., Cetinkaya O., Koca C., Ozger M. (2018). Internet of Hybrid Energy Harvesting Things. IEEE Int. Things J..

[14] Zhou G., Huang L., Li W., Zhu Z. (2014). Harvesting Ambient Environmental Energy for Wireless Sensor Networks: A Survey. J. Sens..

[15] Gomes T., Brito J., Abreu H., Gomes H., Cabral J. GreenMon: An efficient wireless sensor network monitoring solution for greenhouses. Proceedings of the 2015 IEEE International Conference on Industrial Technology (ICIT).

[16] Viñolo C., Toma D., Mànuel A., del Rio J. Sea motion electrical energy generator for low-power applications. Proceedings of the 2013 MTS/IEEE OCEANS.

[17] Weddell A., Merrett G.V., Harris N., Al-Hashimi B.M. (2008). Energy Harvesting and Management for Wireless Autonomous Sensors. Meas. Control.

[18] Li K., Shu L., Mukherjee M., Wang D., Hu L. Prolonging Network Lifetime with Sleep Scheduling for Solar Harvesting Industrial WSNs. Proceedings of the 2016 IEEE 18th International Conference on High Performance Computing and Communications; IEEE 14th International Conference on Smart City; IEEE 2nd International Conference on Data Science and Systems (HPCC/SmartCity/DSS).

[19] Niyato D., Hossain E., Fallahi A. (2007). Sleep and Wakeup Strategies in Solar-Powered Wireless Sensor/Mesh Networks: Performance Analysis and Optimization. IEEE Trans. Mob. Comput..

[20] Mukherjee M., Shu L., Prasad R.V., Wang D., Hancke G.P. (2019). Sleep Scheduling for Unbalanced Energy Harvesting in Industrial Wireless Sensor Networks. IEEE Commun. Mag..

[21] Chen H., Li X., Zhao F. (2016). A Reinforcement Learning-Based Sleep Scheduling Algorithm for Desired Area Coverage in Solar-Powered Wireless Sensor Networks. IEEE Sens. J..

[22] Sharma S., Kumar Jain K., Sharma A. (2015). Solar Cells: In Research and Applications—A Review. Mater. Sci. Appl..

[23] Yan J., Saunders B.R. (2014). Third-generation solar cells: A review and comparison of polymer:fullerene, hybrid polymer and perovskite solar cells. RSC Adv..

[24] Conibeer G. (2007). Third-generation photovoltaics. Mater. Today.

[25] Xi H., Chen D., Lv L., Zhong P., Lin Z., Chang J., Wang H., Wang B., Ma X., Zhang C. (2017). High performance transient organic solar cells on biodegradable polyvinyl alcohol composite substrates. RSC Adv..

[26] Chen S., Yao H., Hu B., Zhang G., Arunagiri L., Ma L.K., Huang J., Zhang J., Zhu Z., Bai F. (2018). A Nonfullerene Semitransparent Tandem Organic Solar Cell with 10.5% Power Conversion Efficiency. Adv. Energy Mater..

[27] Wang W.S., O’Donnell T., Ribetto L., O’Flynn B., Hayes M., O’Mathuna C. Energy harvesting embedded wireless sensor system for building environment applications. Proceedings of the 2009 1st International Conference on Wireless Communication, Vehicular Technology, Information Theory and Aerospace Electronic Systems Technology.

[28] Berger A., Hörmann L.B., Leitner C., Oswald S.B., Priller P., Springer A. Sustainable energy harvesting for robust wireless sensor networks in industrial applications. Proceedings of the 2015 IEEE Sensors Applications Symposium (SAS).

[29] Weddell A.S., Merrett G.V., Al-Hashimi B.M. (2012). Photovoltaic sample-and-hold circuit enabling MPPT indoors for low-power systems. IEEE Trans. Circuits Syst. Regul. Pap..

[30] Sharma H., Haque A., Jaffery Z.A. (2018). Modeling and Optimisation of a Solar Energy Harvesting System for Wireless Sensor Network Nodes. J. Sens. Actuator Netw..

[31] Kim Y., Evans R.G., Iversen W.M. (2008). Remote Sensing and Control of an Irrigation System Using a Distributed Wireless Sensor Network. IEEE Trans. Instrum. Meas..

[32] Martinez K., Ong R., Hart J. Glacsweb: A sensor network for hostile environments. Proceedings of the 2004 First Annual IEEE Communications Society Conference on Sensor and Ad Hoc Communications and Networks, 2004. IEEE SECON 2004.

[33] Pérez C.A., Jiménez M., Soto F., Torres R., López J.A., Iborra A. A system for monitoring marine environments based on Wireless Sensor Networks. Proceedings of the OCEANS 2011 IEEE.

[34] Bader S., Oelmann B. Enabling Battery-Less Wireless Sensor Operation Using Solar Energy Harvesting at Locations with Limited Solar Radiation. Proceedings of the Fourth International Conference on Sensor Technologies and Applications.

[35] Brunelli D., Moser C., Thiele L., Benini L. (2009). Design of a Solar-Harvesting Circuit for Batteryless Embedded Systems. IEEE Trans. Circuits Syst. Regul. Pap..

[36] UoS Lora Nodes LoRaWAN Node development at University of Southampton. https://github.com/loranodes/.

[37] Habibzadeh M., Hassanalieragh M., Ishikawa A., Soyata T., Sharma G. (2017). Hybrid Solar-Wind Energy Harvesting for Embedded Applications: Supercapacitor-Based System Architectures and Design Tradeoffs. IEEE Circuits Syst. Mag..

[38] Nasiri A., Zabalawi S.A., Mandic G. (2009). Indoor Power Harvesting Using Photovoltaic Cells for Low-Power Applications. IEEE Trans. Ind. Electron..

[39] Tan Y.K., Panda S.K. (2011). Energy Harvesting From Hybrid Indoor Ambient Light and Thermal Energy Sources for Enhanced Performance of Wireless Sensor Nodes. IEEE Trans. Ind. Electron..

[40] Alshehri A., Parrott B., Outa A., Amer A., Abdellatif F., Trigui H., Carrasco P., Patel S., Taie I. Dust mitigation in the desert: Cleaning mechanisms for solar panels in arid regions. Proceedings of the 2014 Saudi Arabia Smart Grid Conference (SASG).

[41] Mazumder M., Horenstein M.N., Stark J.W., Girouard P., Sumner R., Henderson B., Sadder O., Hidetaka I., Biris A.S., Sharma R. (2013). Characterization of Electrodynamic Screen Performance for Dust Removal from Solar Panels and Solar Hydrogen Generators. IEEE Trans. Ind. Appl..

[42] Strik D.P., Timmers R.A., Helder M., Steinbusch K.J., Hamelers H.V., Buisman C.J. (2011). Microbial solar cells: Applying photosynthetic and electrochemically active organisms. Trends Biotechnol..

[43] Rosenbaum M., He Z., Angenent L.T. (2010). Light energy to bioelectricity: Photosynthetic microbial fuel cells. Curr. Opin. Biotechnol..

[44] Yoon S., Lee H., Fraiwan A., Dai C., Choi S. (2014). A Microsized Microbial Solar Cell: A demonstration of photosynthetic bacterial electrogenic capabilities. IEEE Nanatechnol. Mag..

[45] Lam K.B., Chiao M., Lin L. A micro photosynthetic electrochemical cell. Proceedings of the Sixteenth Annual International Conference on Micro Electro Mechanical Systems.

[46] Chiao M., Lam K.B., Lin L. (2006). Micromachined microbial and photosynthetic fuel cells. J. Micromech. Microeng..

[47] Ramanan A.V., Pakirisamy M., Williamson S.S. (2015). Advanced Fabrication, Modeling, and Testing of a Microphotosynthetic Electrochemical Cell for Energy Harvesting Applications. IEEE Trans. Power Electron..

[48] Strik D.P., Terlouw H., Hamelers H.V.M., Buisman C.J.N. (2008). Renewable sustainable biocatalyzed electricity production in a photosynthetic algal microbial fuel cell (PAMFC). Appl. Microbiol. Biotechnol..

[49] Petäjäjärvi J., Kaleva J., Mikhaylov K., Pulkkinen H., Ahola J., Björkgren M. Automatic charging of an energy harvesting powered sensor node from controllable energy source. Proceedings of the 2018 14th International Wireless Communications Mobile Computing Conference (IWCMC).

[50] Helmers H., Bett A.W. Photovoltaic laser power converters for wireless optical power supply of sensor systems. Proceedings of the 2016 IEEE International Conference on Wireless for Space and Extreme Environments (WiSEE).

[51] Liu C., Chen P., Li K. (2014). A 500 W low-temperature thermoelectric generator: Design and experimental study. Int. J. Hydrogen Energy.

[52] DiSalvo F.J. (1999). Thermoelectric Cooling and Power Generation. Science.

[53] Ilahi T., Abid M., Ilahi T. Design and analysis of thermoelectric material based roof top energy harvesting system for Pakistan. Proceedings of the 2015 Power Generation System and Renewable Energy Technologies (PGSRET).

[54] Dias P.C., Morais F.J.O., de Morais França M.B., Ferreira E.C., Cabot A., Dias J.A.S. (2015). Autonomous Multisensor System Powered by a Solar Thermoelectric Energy Harvester With Ultralow-Power Management Circuit. IEEE Trans. Instrum. Meas..

[55] Carvalhaes-Dias P., Cabot A., Siqueira Dias J.A. (2018). Evaluation of the Thermoelectric Energy Harvesting Potential at Different Latitudes Using Solar Flat Panels Systems with Buried Heat Sink. Appl. Sci..

[56] Verma G., Sharma V. (2019). A Novel Thermoelectric Energy Harvester for Wireless Sensor Network Application. IEEE Trans. Ind. Electron..

[57] Prijić A., Vračar L., Vučković D., Milić D., Prijić Z. (2015). Thermal Energy Harvesting Wireless Sensor Node in Aluminum Core PCB Technology. IEEE Sens. J..

[58] Wang Z., Leonov V., Fiorini P., Hoof C.V. (2009). Realization of a wearable miniaturized thermoelectric generator for human body applications. Sens. Actuators A.

[59] Leonov V., Torfs T., Fiorini P., Hoof C.V. (2007). Thermoelectric Converters of Human Warmth for Self-Powered Wireless Sensor Nodes. IEEE Sens. J..

[60] Jänsch D., Lauterbach J., Pohle M., Steinberg P. Thermoelectrics—An Opportunity for the Automotive Industry?. Proceedings of the International Conference of Energy and Thermal Management, Air Conditioning, Waste Heat Recovery.

[61] Orr B., Akbarzadeh A., Mochizuki M., Singh R. (2016). A review of car waste heat recovery systems utilising thermoelectric generators and heat pipes. Appl. Therm. Eng..

[62] Legros A., Guillaume L., Diny M., Zaïdi H., Lemort V. (2014). Comparison and Impact of Waste Heat Recovery Technologies on Passenger Car Fuel Consumption in a Normalized Driving Cycle. Energies.

[63] Rajoo S., Romagnoli A., Ricardo M.B., Pesyridis A., Copeland C., Bin Mamat A. (2014). Automotive Exhaust Waste Heat Recovery Technologies.

[64] Program N.T.T. Pyroelectric Sandwich Thermal Energy Harvester. https://technology.nasa.gov/patent/LAR-TOPS-221.

[65] Sebald G., Guyomar D., Agbossou A. (2009). On thermoelectric and pyroelectric energy harvesting. Smart Mater. Struct..

[66] Cha G., Jia Y., Ju Y.S. High-power density pyroelectric energy harvesters incorporating switchable liquid-based thermal interfaces. Proceedings of the 2012 IEEE 25th International Conference on Micro Electro Mechanical Systems (MEMS).

[67] Ravindran S.K.T., Huesgen T., Kroener M., Woias P. A self-sustaining pyroelectric energy harvester utilizing spatial thermal gradients. Proceedings of the 2011 16th International Solid-State Sensors, Actuators and Microsystems Conference.

[68] Sultana A., Alam M.M., Middya T.R., Mandal D. (2018). A pyroelectric generator as a self-powered temperature sensor for sustainable thermal energy harvesting from waste heat and human body heat. Appl. Energy.

[69] Zheng H., Zi Y., He X., Guo H., Lai Y.C., Wang J., Zhang S.L., Wu C., Cheng G., Wang Z.L. (2018). Concurrent Harvesting of Ambient Energy by Hybrid Nanogenerators for Wearable Self-Powered Systems and Active Remote Sensing. ACS Appl. Mater. Interfaces.

[70] Mart C., Weinreich W., Czernohorsky M., Riedel S., Zybell S., Kuhnel K. CMOS Compatible Pyroelectric Applications Enabled by Doped HfO2Films on Deep-Trench Structures. Proceedings of the 2018 48th European Solid-State Device Research Conference (ESSDERC).

[71] Bennett G.L., Skrabek E.A. Power performance of US space radioisotope thermoelectric generators. Proceedings of the Fifteenth International Conference on Thermoelectrics, ICT ’96.

[72] Bass J.C., Allen D.T. Milliwatt radioisotope power supply for space applications. Proceedings of theEighteenth International Conference on Thermoelectrics, ICT’99 (Cat. No.99TH8407).

[73] Lange R.G., Carroll W.P. (2008). Review of recent advances of radioisotope power systems. Energy Convers. Manag..

[74] Stapfer G., Truscello V.C. The long-term performance degradation of a radioisotope thermoelectric generator using silicon germanium. Proceedings of the Eleventh Intersociety Energy Conversion Engineering Conference.

[75] Huffman F.N., Migliore J.J., Robinson W.J., Norman J.C. (1974). Radioisotope powered cardiac pacemakers. Cardiovasc. Dis..

[76] Mallela V.S., Ilankumaran V., Rao N.S. (2004). Trends in cardiac pacemaker batteries. Indian Pacing Electrophysiol. J..

[77] Ashraf M., Masoumi N. (2016). A Thermal Energy Harvesting Power Supply with an Internal Startup Circuit for Pacemakers. IEEE Trans. Very Large Scale Integr. VLSI Syst..

[78] Cockfield R.D., Chan T.S. Stirling radioisotope generator for Mars surface and deep space missions. Proceedings of the 2002 IECEC 37th Intersociety Energy Conversion Engineering Conference.

[79] Chen Y., Twigg C.M., Sadik O.A., Tong S. A self-powered adaptive wireless sensor network for wastewater treatment plants. Proceedings of the 2011 IEEE International Conference on Pervasive Computing and Communications Workshops (PERCOM Workshops).

[80] Richter K.E., George R., Hardy K. Autonomous, retrievable, deep sea microbial fuel cell. Proceedings of the OCEANS 2015—Genova.

[81] Zhang D., Ge Y., Wang W. Study of a terrestrial microbial fuel cell and its power generation performance. Proceedings of the 2013 IEEE International Conference of IEEE Region 10 (TENCON 2013).

[82] Sun G., Qiao G., Zhao L., Chen Z. (2013). Events as Power Source: Wireless Sustainable Corrosion Monitoring. Sensors.

[83] Sun G., Qiao G., Xu B. (2011). Corrosion Monitoring Sensor Networks with Energy Harvesting. IEEE Sens. J..

[84] Qiao G., Hong Y., Sun G., Yang O. (2013). Corrosion Energy: A Novel Source to Power the Wireless Sensor. IEEE Sens. J..

[85] Ouellette S.A., Todd M.D. (2014). Cement Seawater Battery Energy Harvester for Marine Infrastructure Monitoring. IEEE Sens. J..

[86] Holmes A.S., Hong G., Pullen K.R., Buffard K.R. Axial-flow microturbine with electromagnetic generator: design, CFD simulation, and prototype demonstration. Proceedings of the 17th IEEE International Conference on Micro Electro Mechanical Systems, Maastricht MEMS 2004 Technical Digest.

[87] Carli D., Brunelli D., Bertozzi D., Benini L. A high-efficiency wind-flow energy harvester using micro turbine. Proceedings of the SPEEDAM 2010.

[88] Da Costa E.F., De Oliveira N.E., Morais F.J.O., Carvalhaes-Dias P., Duarte L.F.C., Cabot A., Siqueira Dias J.A. (2017). A Self-Powered and Autonomous Fringing Field Capacitive Sensor Integrated into a Micro Sprinkler Spinner to Measure Soil Water Content. Sensors.

[89] Zhu D., Beeby S., Tudor M., White N., Harris N. (2013). A novel miniature airflow energy harvester for wireless sensing applications in buildings. IEEE Sens. J..

[90] Li S., Lipson H. Vertical-Stalk Flapping-Leaf Generator for Wind Energy Harvesting. Proceedings of the ASME 2009 Conference on Smart Materials, Adaptive Structures and Intelligent Systems SMASIS2009.

[91] Erturk A., Vieira W.G.R., De Marqui C., Inman D.J. (2010). On the energy harvesting potential of piezoaeroelastic systems. Appl. Phys. Lett..

[92] Matova S., Elfrink R., Vullers R., Schaijk R. (2011). Harvesting energy from airflow with a michromachined piezoelectric harvester inside a Helmholtz resonator. J. Micromech. Microeng..

[93] Blystad L.J., Halvorsen E., Husa S. (2010). Piezoelectric MEMS energy harvesting systems driven by harmonic and random vibrations. IEEE Trans. Ultrason. Ferroelectr. Freq. Control.

[94] Pan C., Zhang J., Xia H., Yu L. A piezoelectric wind energy harvesting device with right-angle cantilever beam. Proceedings of the 2017 Symposium on Piezoelectricity, Acoustic Waves, and Device Applications (SPAWDA).

[95] Kok S.L., White N.M., Harris N.R. A Free-Standing, Thick-Film Piezoelectric Energy Harvester. Proceedings of the IEEE Sensors 2008.

[96] Harris N., Beeby S., Tudor J., Zhu D. Thick-film Piezoelectric Vibration Harvesting –A HUMS Application. Proceedings of the APCOT2010.

[97] Rocha J.G., Goncalves L.M., Rocha P.F., Silva M.P., Lanceros-Mendez S. (2010). Energy Harvesting From Piezoelectric Materials Fully Integrated in Footwear. IEEE Trans. Ind. Electron..

[98] Khaligh A., Zeng P., Zheng C. (2010). Kinetic Energy Harvesting Using Piezoelectric and Electromagnetic Technologies—State of the Art. IEEE Trans. Ind. Electron..

[99] Elhalwagy A.M., Ghoneem M.Y.M., Elhadidi M. (2017). Feasibility Study for Using Piezoelectric Energy Harvesting Floor in Buildings’ Interior Spaces. Energy Procedia.

[100] Choi Y.M., Lee M.G., Jeon Y. (2017). Wearable Biomechanical Energy Harvesting Technologies. Energies.

[101] Wong C.H., Dahari Z., Abd Manaf A., Miskam M.A. (2015). Harvesting Raindrop Energy with Piezoelectrics: A Review. J. Electron. Mater..

[102] Guigon R., Chaillout J.J., Jager T., Despesse G. (2008). Harvesting raindrop energy: Experimental study. Smart Mater. Struct..

[103] Chua K., Hor Y.F., Lim H.C. (2016). Raindrop Kinetic Energy Piezoelectric Harvesters and Relevant Interface Circuits: Review, Issues and Outlooks. Sens. Transducers.

[104] Ong Z.Z., Wong V.K., Ho J.H. (2016). Performance enhancement of a piezoelectric rain energy harvester. Sens. Actuators A.

[105] Ali G., Wagner J., Moline D., Schweisinger T. (2015). Energy harvesting from atmospheric variations—Theory and test. Renewable Energy.

[106] Zhao C., Yisrael S., Smith J.R., Patel S.N. Powering Wireless Sensor Nodes with Ambient Temperature Changes. Proceedings of the 2014 ACM International Joint Conference on Pervasive and Ubiquitous Computing.

[107] Ganesh S., Ali G., Moline D., Schweisinger T., Wagner J. (2018). Conversion of atmospheric variations into electric power—Design and analysis of an electric power generator system. Renewable Energy.

[108] Jefimenko O.D., Walker D.K. (1978). Electrostatic Current Generator Having a Disk Electret as an Active Element. IEEE Trans. Ind. Appl..

[109] Boisseau S., Despesse G., Seddik B.A. (2012). Electrostatic Conversion for Vibration Energy Harvesting. arXiv.

[110] Sterken T., Fiorini P., Altena G., Van Hoof C., Puers R. Harvesting energy from vibrations by a micromachined electret generator. Proceedings of the TRANSDUCERS 2007—2007 International Solid-State Sensors, Actuators and Microsystems Conference.

[111] Crovetto A., Wang F., Hansen O. (2014). Modeling and Optimization of an Electrostatic Energy Harvesting Device. J. Microelectromech. Syst..

[112] Logothetis I., Vassiliadis S., Siores E. (2017). Triboelectric effect in energy harvesting. IOP Conf. Ser. Mater. Sci. Eng..

[113] Yang W., Chen J., Zhu G., Yang J., Bai P., Su Y., Jing Q., Cao X., Wang Z.L. (2013). Harvesting Energy from the Natural Vibration of Human Walking. ACS Nano.

[114] Kim S., Vyas R., Bito J., Niotaki K., Collado A., Georgiadis A., Tentzeris M.M. (2014). Ambient RF Energy-Harvesting Technologies for Self-Sustainable Standalone Wireless Sensor Platforms. Proc. IEEE.

[115] Nalini M., Kumar J.V.N., Kumar R.M., Vignesh M. Energy harvesting and management from ambient RF radiation. Proceedings of the 2017 International Conference on Innovations in Green Energy and Healthcare Technologies (IGEHT).

[116] Lim T.B., Lee N.M., Poh B.K. Feasibility study on ambient RF energy harvesting for wireless sensor network. Proceedings of the 2013 IEEE MTT-S International Microwave Workshop Series on RF and Wireless Technologies for Biomedical and Healthcare Applications (IMWS-BIO).

[117] Tran H., Kaddoum G., Truong K.T. (2018). Resource Allocation in SWIPT Networks Under a Nonlinear Energy Harvesting Model: Power Efficiency, User Fairness, and Channel Nonreciprocity. IEEE Trans. Veh. Technol..

[118] Brown W.C. (1980). The History of the Development of the Rectenna. NASA. Johnson Space Center Solar Power Satellite Microwave Transmission and Reception.

[119] Xie L., Shi Y., Hou Y.T., Lou A. (2013). Wireless power transfer and applications to sensor networks. IEEE Wirel. Commun..

[120] Powercast’s Wireless Charging Technology Available Globally from Digi-Key. https://www.powercastco.com/powercasts-wireless-charging-technology-available-globally-digi-key/.

[121] P2110B | 915Mhz RF Powerharvester Receiver. https://www.powercastco.com/wp-content/uploads/2016/12/P2110B-Datasheet-Rev-3.pdf.

[122] Zungeru A.M., Ang L.M., Prabaharan S.R.S., Phooi Seng K. (2012). Radio Frequency Energy Harvesting and Management for Wireless Sensor Networks. arXiv.

[123] Valenta C.R., Durgin G.D. (2014). Harvesting Wireless Power: Survey of Energy-Harvester Conversion Efficiency in Far-Field, Wireless Power Transfer Systems. IEEE Microwave Mag..

[124] Lee T., Kennedy H.R.B., Bodnar R.A., Redman-White W. A CMOS MF energy harvesting and data demodulator receiver for wide area low duty cycle applications with 230 mV start-up voltage. Proceedings of the 2016 IEEE Nordic Circuits and Systems Conference (NORCAS).

[125] Kurs A., Karalis A., Moffatt R., Joannopoulos J.D., Fisher P., Soljačić M. (2007). Wireless Power Transfer via Strongly Coupled Magnetic Resonances. Science.

[126] Jiang H., Zhang J., Lan D., Chao K.K., Liou S., Shahnasser H., Fechter R., Hirose S., Harrison M., Roy S. (2013). A Low-Frequency Versatile Wireless Power Transfer Technology for Biomedical Implants. IEEE Trans. Biomed. Circuits Syst..

[127] WiTricity. http://witricity.com/.

[128] Ho S.L., Wang J., Fu W.N., Sun M. (2011). A Comparative Study Between Novel Witricity and Traditional Inductive Magnetic Coupling in Wireless Charging. IEEE Trans. Magn..

[129] WiTricity | Automotive Solutions. http://witricity.com/products/automotive/.

[130] Ishida H., Kyoden T., Furukawa H. (2018). Super-low-frequency wireless power transfer with lightweight coils for passing through a stainless steel plate. Rev. Sci. Instrum..

[131] Fakih A., Rais U., Maniar S., Naik R., Siddiqui H. Implementation of a Self-Sustainable Infrastructure with remote power management. Proceedings of the 2018 International Conference on Smart City and Emerging Technology (ICSCET).

[132] Mane P., Xie J., Leang K.K., Mossi K. (2011). Cyclic energy harvesting from pyroelectric materials. IEEE Trans. Ultrason. Ferroelectr. Freq. Control.

[133] Pietrelli A., Ferrara V., Micangeli A., Uribe L. Efficient energy harvesting for Microbial Fuel Cell dedicated to Wireless Sensor Network. Proceedings of the 2015 XVIII AISEM Annual Conference.

[134] Boisseau S., Despesse G., Ricart T., Defay E., Sylvestre A. (2011). Cantilever-based electret energy harvester. Smart Mater. Struct..

[135] Worgan P., Clare L., Proynov P., H Stark B., Coyle D. Inductive Power Transfer for On-body Sensors: Defining a design space for safe, wirelessly powered on-body health sensors. Proceedings of the 2015 9th International Conference on Pervasive Computing Technologies for Healthcare (PervasiveHealth).

[136] Deng F., Yue X., Fan X., Guan S., Xu Y., Chen J. (2019). Multisource Energy Harvesting System for a Wireless Sensor Network Node in the Field Environment. IEEE Internet Things J..

[137] Wang C., Li J., Yang Y., Ye F. (2018). Combining Solar Energy Harvesting with Wireless Charging for Hybrid Wireless Sensor Networks. IEEE Trans. Mob. Comput..

[138] Zhu Y., Li E., Chi K. (2018). Encoding Scheme to Reduce Energy Consumption of Delivering Data in Radio Frequency Powered Battery-Free Wireless Sensor Networks. IEEE Trans. Veh. Technol..

[139] Esteves V., Antonopoulos A., Kartsakli E., Puig-Vidal M., Miribel-Català P., Verikoukis C. (2015). Cooperative energy harvesting-adaptive MAC protocol for WBANs. Sensors.

[140] Ibarra E., Antonopoulos A., Kartsakli E., Rodrigues J.J.P.C., Verikoukis C. (2016). QoS-Aware Energy Management in Body Sensor Nodes Powered by Human Energy Harvesting. IEEE Sens. J..

